# The environmental awareness of nurses as environmentally sustainable health care leaders: a mixed method analysis

**DOI:** 10.1186/s12912-024-01895-z

**Published:** 2024-04-03

**Authors:** Olga María Luque-Alcaraz, Pilar Aparicio-Martínez, Antonio Gomera, Manuel Vaquero-Abellán

**Affiliations:** 1 Neurosurgery Department, Hospital Universitario Reina Sofía, Andalusian Health Care System, 14071 Cordoba, Spain; 2 Environmental Protection Office (SEPA), University of Córdoba, Campus Rabanales, 14014 Córdoba, Spain; 3 GE 10 Research Groups of Clinical-Epidemiological Research in Primary Care, University Biomedical Program for Occupational Medicine, Occupational Epidemiology and Sustainability, Maimonides Institute of Biomedicine of Cordoba (IMIBIC), 14071 Cordoba, Spain; 4 Nursing, Pharmacology and Physiotherapy Department, Faculty of Medicine and Nursing, University of Cordoba, 14071 Cordoba, Spain; 5 GA16 Lifestyles, Innovation and Health, Maimonides Institute of Biomedicine of Cordoba (IMIBIC), 14071 Cordoba, Spain

**Keywords:** Nurses, Environmental health, Attitude of health personnel, Sustainable development, Health Knowledge, attitudes, and practices, Organizational Culture, Leadership

## Abstract

**Background:**

People worldwide are concerned with the possibility of climate change, microplastics, air pollution, and extreme weather affecting human health. Countries are implementing measures to reduce environmental impacts. Nurses play a vital role, primarily through Green Teams, in the process of promoting sustainable practices and minimizing the environmental footprint of health care facilities. Despite existing knowledge on this topic, assessing nurses’ environmental awareness and behavior, including the barriers they face, is crucial with regard to improving sustainable health care practices.

**Aim:**

To analyze the environmental awareness and behavior of nurses, especially nurse leaders, as members of the Green Team and to identify areas for improvement with regard to the creation of a sustainable environment.

**Methods:**

A sequential mixed-method study was conducted to investigate Spanish nurses. The study utilized an online survey and interviews, including participant observation. An online survey was administered to collect quantitative data regarding environmental awareness and behavior. Qualitative interviews were conducted with environmental nurses in specific regions, with a focus on Andalusia, Spain.

**Results:**

Most of the surveyed nurses (*N* = 314) exhibited moderate environmental awareness (70.4%), but their environmental behavior and activities in the workplace were limited (52.23% of participants rarely performed relevant actions, and 35.03% indicated that doing so was difficult). Nurses who exhibited higher levels of environmental awareness were more likely to engage in sustainable behaviors such as waste reduction, energy conservation, and environmentally conscious purchasing decisions (*p* < 0.05). Additionally, the adjusted model indicated that nurses’ environmental behavior and activities in the workplace depend on the frequency of their environmental behaviors outside work as well as their sustainable knowledge (*p* < 0.01). The results of the qualitative study (*N* = 10) highlighted certain limitations in their daily practices related to environmental sustainability, including a lack of time, a lack of bins and the pandemic. Additionally, sustainable environmental behavior on the part of nursing leadership and the Green Team must be improved.

**Conclusions:**

This study revealed that most nurses have adequate knowledge, attitudes, and behaviors related to environmental sustainability both inside and outside the workplace. Limitations were associated with their knowledge and behaviors outside of work. This study also highlighted the barriers and difficulties that nurses face in their attempts to engage in adequate environmental behaviors in the workplace. Based on these findings, interventions led by nurses and the Green Team should be developed to promote sustainable behaviors among nurses and address the barriers and limitations identified in this research.

**Graphical Abstract:**

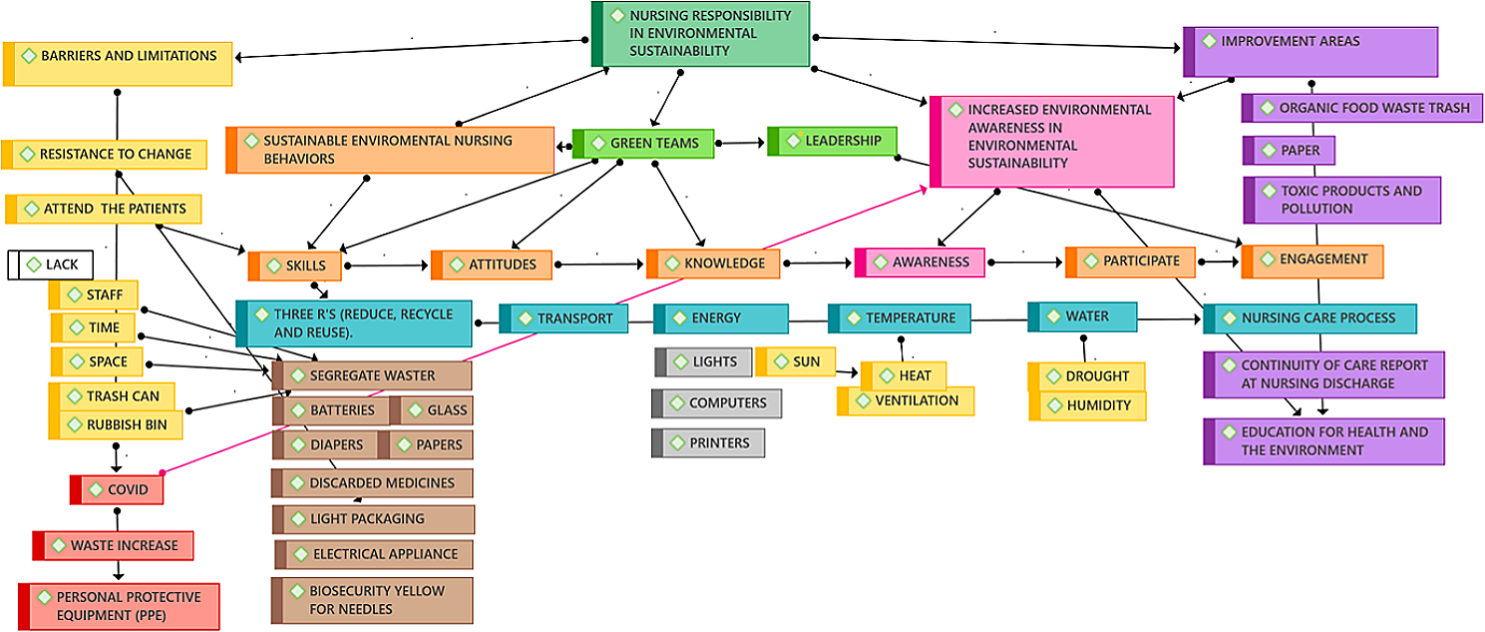

**Supplementary Information:**

The online version contains supplementary material available at 10.1186/s12912-024-01895-z.

## Introduction

The impact of climate change on human society is a global concern, especially with regard to microplastics, resource shortages, air pollution, droughts, and extreme weather. Such consequences affect human health both directly and indirectly, resulting in an increase in pathologies and a deterioration in medical attention [1, 2]. In this context, diverse measures aimed at reducing the environmental impact of daily activities and minimizing the ecological footprint thereof [3] have been implemented by multiple countries [4–7]; these activities have been framed as environmental regulations in line with the Sustainable Development Goals (SDGs) [8].

The SDGs are being integrated into governments and a variety of other contexts, including the health care system. Spain is dedicated to such a goal, i.e., that of promoting a greener and more democratic health care transition. To achieve this goal, strategic plans have been developed to mitigate the effects of climate change [9, 10]. One specific such program is the Strategic Health and Environment Plan (PESMA) [11], whose aim is to enhance the synergy between health and the environment innovatively by assessing the impact of the population in terms of 14 environmental indicators [12].

One such indicator focuses on the resources and support needed for sustainable practices, especially for nurses, due to the impact of the environment on their work [13, 14]. The PESMA highlights the fact that health care providers should be included in strategies to reduce carbon footprints, build resilience to address the challenges associated with climate change and embrace a leadership role in the task of promoting sustainable health care practices [13–16]. Another critical aspect of PESMA focuses on education, training, and incentives that can promote sustainable behavior among health care workers, especially nurses [17, 18]. As frontline health care workers, nurses have a unique opportunity to advocate for sustainable practices and reduce the environmental impact of the health care system. Nurses’ knowledge and behavior are limited despite the fact that nurses have positive attitudes toward environmental sustainability [19].

This situation stands in contrast to the role of nurses in the creation of more sustainable hospitals via the “Green Team” [20]. The Green Team, which originated in the United States of America a decade ago, is a committee that is responsible for finding and implementing sustainability projects to decrease the environmental impacts of daily operations. Members of various departments collaborate with sustainability staff to detect opportunities, spread awareness, and promote staff involvement in line with the Committee’s mission [21]. The team, which typically consists of and is led by nurses, aims to increase awareness of the health care industry’s effect on the environment and to develop tactics to mitigate the adverse environmental effects of hospitals.

In Spain, Green Teams, which span multiple disciplines and usually led by nursing professionals, are committed to sustainable change in health care [22]. Environmental nursing leaders on Green Teams control environmental sustainability in health care settings and provide education, resources, and support to other professionals with regard to the implementation of sustainable practices [23]. Accordingly, all nurses can contribute to the tasks of mitigating the impact of climate change on public health outcomes and promoting sustainable health for all [24]. These actions improve nurses’ knowledge, attitudes, and behavior in terms of sustainability and promote sustainable practices in health care settings, thus leading to a better understanding of the barriers faced by nurses in this context [24–26].

However, measuring and identifying nurses’ environmental awareness is essential for the promotion of sustainable hospitals [27, 28]. Multidimensional indicators have been proposed for this purpose [16], the responsibility for which lies with nurse leaders on Green Teams. Nurses are responsible for promoting sustainability in health care organizations, as discussed by Kallio et al. (2018) [29], as well as for promoting nursing competencies related to environmental sustainability [30]. Several studies, including Harris et al. (2009) and Phiri et al. (2022), have examined nurses’ roles in environmental health and the effects of their leadership on the promotion of sustainability, especially during the COVID-19 pandemic, thereby emphasizing the importance of leadership [31, 32].

As Ojemeni et al. (2019) discussed, leadership effectiveness in Green Teams, nursing teams and health care organizations must prioritize quality control and health care improvement to ensure sustainable development [33].

The topic of environmental management in health care organizations has been studied extensively, and an environmental or ecological model of care for promoting sustainability has been proposed [34]. As environmental creators and leaders on Green Teams, nurses are vital for minimizing hazardous waste in health care settings and improving awareness [35].

Although nurses have some degree of existing knowledge and awareness of sustainability, it is crucial to assess their proficiency in environmental matters and to gauge their environmental awareness. Such an evaluation can help identify areas for improvement within clinical management units [20, 33, 36]. Education and training programs can effectively promote sustainable behavior among nurses, but interventions should also address the barriers they face in their attempts to implement sustainable practices [37]. Therefore, it is imperative to examine the factors that foster sustainable behavior among nurses and to identify effective interventions that can promote sustainable health care practices and minimize the environmental footprint of health care facilities. Accordingly, this study aimed to analyze the environmental awareness and behavior of nurses, especially nurse leaders, as members of the Green Team and to identify areas for improvement with regard to creating a sustainable environment.

## Methods

### Study design

A sequential mixed-method study was conducted based on an online survey and interviews with a representative sample of Spanish nurses, including participant observation.

The study was divided into two phases. In the first phase, a cross-sectional, descriptive exploratory analysis was performed; this analysis relied on the results revealed using the Nurse’s Environmental Awareness Tool in Spanish (NEAT-es) [38], which was divided into three subscales: nursing awareness scale (NAS), environmental behaviors outside the workplace (PEB) and sustainable behaviors in the workplace (NPEB). In the second phase, qualitative interviews with environmental nurses (see Supplementary file 1) were conducted in regions featuring specific environmental units that were available in person (Andalusia).

### Participants

The participants were recruited from public and private institutions associated with the National Health System, particularly from the nursing staff. The scope of the study focused on Spain, and the sample included all the nursing staff who completed the questionnaire and met the inclusion criteria.

The sampling process focused on the population of nurses in Spain in 2020, which was estimated to consist of 388,153 nurses. Therefore, a random sample of 314 participating individuals was sufficient to estimate the population with 95% confidence and an accuracy of +/- 2% units, which was expected to account for approximately 90% of the overall population. The inclusion and exclusion criteria used for the sample focused on nursing staff, nursing care auxiliary technicians, and students with relevant degrees, as this members of this group have the most significant presence in the health system and engage in direct and daily contact with environmental management in health centers (hospitals, primary care centers, sociosanitary centers and others). The remaining health and nonhealth personnel were excluded.

Additionally, the person from each unit who served as the environmental coordinator and other nurses from the ward who were members of the Green Team were asked to participate in the interviews and observations. The environmental coordinators, most of who were nursing supervisors, were determined based on the number of members of the Green Team and the sampling calculation used for the observational study. The interviews took place after various sessions, talks, or courses pertaining to environmental sustainability at the clinical management units.

### Data collection

An intentional sampling process was implemented, and the data collection period spanned from November 2019 to March 2021. The observational data were collected in Spain via messages and posts on social media with the goal of quantifying nurses’ environmental awareness.

The initial sample of qualitative study included five environmental nursing leaders (NLs), 14 registered nurses (RNs), and ten nursing undergraduates. The final sample was reduced when the interviews reached data saturation (*N* = 10, five NLs, and five RNs). Before the interviews, a focal group composed of one nurse, one physician, two engineers and a psychologist was tested using the questions included in this research as part of a pilot study (Supplementary file 1). These interviews were conducted at the beginning of the participant’s shift, usually in the morning, and they featured a median time of 30 min, a minimum of 20 min and a maximum of one hour per participant.

One researcher (O.A.L.) also observed nurses during their daily work after the interview from a position within the ward as an added team member or staff member. Nevertheless, the observer did not highlight mistakes or sustainability issues during the observation process. No other researcher was involved in this step of the ethnographic analysis to avoid bias with regard to observing a variety of tasks ranging from preparing medication to implementing treatments.

The data collected through the interviews were recorded on a Samsung Galaxy 31 A, and observations were collected in a field notebook based on the Google Keep and Evernote mobile applications from November 2019 to mid-March 2021. This study was conducted at a regional level 1 hospital in southern Spain, particularly in various clinical management units (neurosurgery, internal medicine, cardiology, traumatology, and COVID-19 units, among others), and it focused on nursing supervisors, who are the leaders who bear responsibility for environmental awareness (NLs), and registered nurses (RNs) who were members of the Green Team.

### Data analysis

The quantitative data were analyzed by reference to descriptive statistics, including the mean, standard deviation (SD), and 95% confidence interval (CI); the relative frequencies of the variables were also analyzed. Normalization tests, Kolmogorov‒Smirnov tests with Lilliefors correction, and Q‒Q tests were used to compare the goodness-of-fit to an average data distribution with regard to continuous or discrete quantitative variables. The comparison of two or three independent means was performed using Student’s t test and analyses of variance for each variable. The Χ^2^ test with Yates’ correction was used to compare percentages and Pearson’s correlation (r) coefficients across the quantitative variables. Finally, associations among the NPEB and the other variables were studied through multiple linear regression. Participant observation was used to support the qualitative study of the reflective ethnographic type [39, 40], and this process ended when the data reached saturation. Two researchers developed transcripts for the interviews based on the recorded interviews and added descriptions based on the notes from the field notebook. The identification of themes and patrons was based on a process of triangulation among the researchers and by cross-checking the results. The interviews with nurses were analyzed to summarize the content analysis and identify keywords and concurrency among the terms. The themes thus identified included Green Teams, sustainable environmental behaviors, environment awareness, leadership barriers and limitations and areas for improvement.

EPIDAT (version 4.2) and SPSS (version 25) software were used to support the quantitative analysis. The computer program ATLAS.ti (version 22) and the Office Package with Microsoft Word Excel (version 2019) were used for the interviews and the visualization of the keywords based on the themes identified based on the records, observations and field notebooks.

## Results

Nurses’ awareness, knowledge, attitudes and skills.

The ages of the Spanish staff, mainly nurses, included in this study (*N* = 314) ranged from 19 to 68, with a mean age of 37.02 ± 12.7, CI = 95%, 35.6–38.4 years); in addition, 76.4% of these participants were women with more than 20 years of working experience (35.1%), and the majority were registered nurses (70.4%). Moreover, 113 (36%) participants worked at a local or regional hospital (30%) and were employees of a public institution (85.3%). Half of the nurses (157) worked only a morning shift (Table 1) in Andalusia, Madrid, or Catalonia (62.4%). The diverse autonomous regions on which this research focused were homogenously distributed and structured in line with the population. The analysis of these areas was also based on the specific inclusion of environmental units led by nurses (Andalusia, Madrid, and Catalonia), in contrast with regions featuring undetermined units or leaders related to this topic (such as Valencia) (37.5%).

**Table 1:** Sociodemographic characteristics of the sample

	Frequencies N %	
	N	%
**Gender**		
Female	240	76.4
Male	74	23.6
Nonbinary	0	0
**Working experience**		
More than 20 years	111	35.4
Between 11 and 20 years	56	17.8
Between 10 and 5 years	43	13.7
Less than 5 years	104	33.1
**Position held**		
Nursing Assistant	18	5.7
Student Nursing Assistant	1	0.3
Undergraduate Nurse	62	19.7
Registered Nurse	221	70.4
Student Nursing Specialist	12	3.9
**Institution**		
Local Hospital	113	36.0
Local Primary Health Care	56	17.8
Regional Hospital	110	35
Regional Primary Health Care	12	3.8
Sociosanitary Institution (i.e., nursing home)	12	3.8
Other	11	3.6
**Character**		
Public	268	85.3
Private	20	6.4
Collaboration between public and private entities	26	8.3
**Shift**		
Only mornings	157	50.0
Only afternoons	14	4.5
Only nights	10	3.2
Rotating shift (switching among other shifts)	131	41.7
Other	2	0.6

**Fig. 1:**
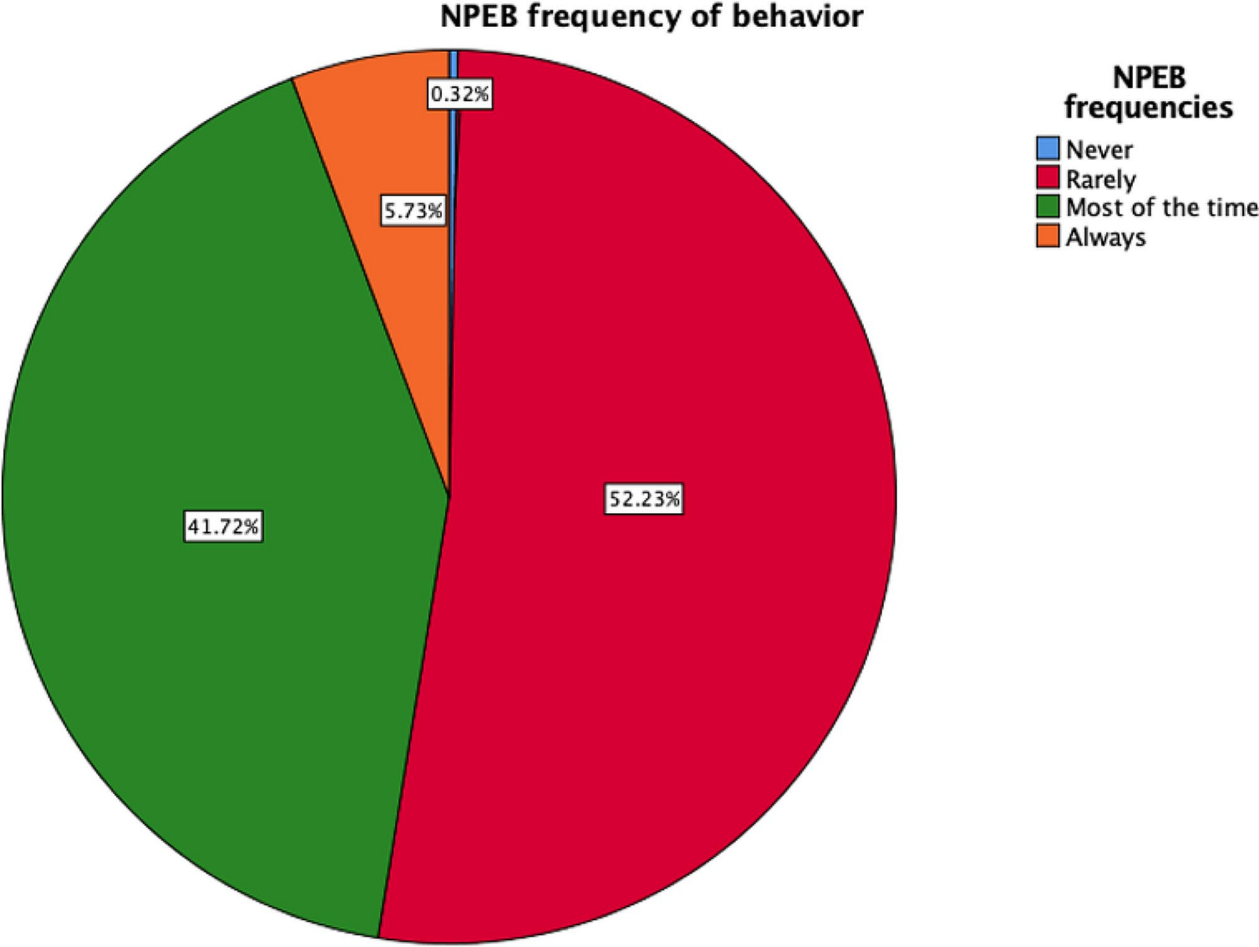
Representation of the frequency of nursing environmental behavior

**Fig. 2:**
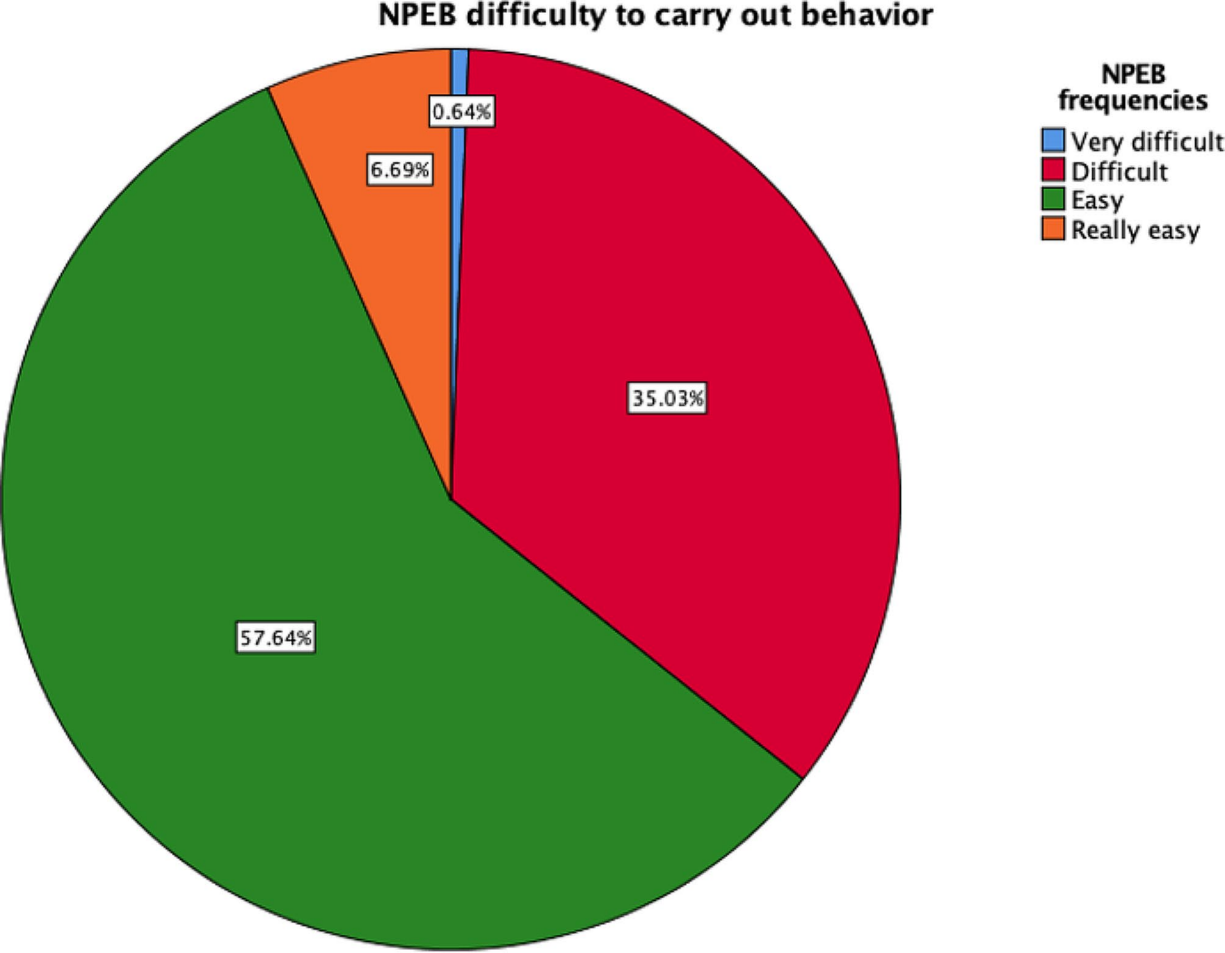
Difficulty of engaging in adequate environmental behaviors

Regarding nursing awareness, nurses scored higher on the PEB (31.83 ± 8.02 CI 95% 30.94–32.72 with regard to frequency vs. 32.36 ± 7.15 CI 95% 31.57–33.15 with respect to difficulty) than on the NAS (26.13 ± 9.91 CI 95% 25.03–27.23 with regard to knowledge vs. 47.39 ± 5.97 CI 95% 46.73–48.05 with respect to impact) and the NPEB (23.82 ± 6.45 CI 95% 23.10-24.53 with regard to frequency vs. 25.71 ± 6.31 CI 95% 25.01–26.41 with respect to difficulty). These results indicated that environmental knowledge among the Spanish population was limited (55.7%), although the nurses included in this research were aware of their potential impact on the environment (70.4%). The PEB subscale focused mostly on following environmental guidelines in their homes (57.3%) because these sustainable domestic tasks are easier for them (63.1%) than tasks in the professional field. The second subscale, NPEB, indicated that sustainable activities such as recycling were easy for the participants (57.6%), but sometimes they engaged in such activities less frequently than they would like (52.2%) (Fig. 1 and Fig. 2).

The sociodemographic variables indicated differences among the NEAT subscales (Table 2). Gender, working experience (with a median value of 10 years), and the position held in the institution and region were relevant with regard to environmental knowledge (*p* < 0.01), environmental behavior outside the workplace (*p* < 0.01), and environmental behavior in the workplace (*p* < 0.01).

**Table 2:** Differences between nurses’ environmental behavior and knowledge

	Differences pertaining to environmental awareness (NAS)		Differences pertaining to environmental behavior outside the workplace (PEB)		Differences pertaining to environmental behavior in the workplace (NPEB)	
	Knowledge	Impact	Frequency	Difficulty	Frequency	Difficulty
**Gender**	0.158	0.004	0.278	0.774	0.031	0. 867
**Working experience**	< 0.001	0.543	< 0.001	0.001	0.001	0.778
**Position held**	0.007	0.710	0.064	0.087	0.002	0.507
**Institution**	0.002	0.476	0.512	0.702	0. 011	0.162
**Character of the institution**	0.272	0.557	0.488	0.903	0. 485	0.949
**Shift**	0.218	0.914	0.121	0.237	0.839	0.277
**Regions featuring nursing leaders**	0.003	0.910	0.055	0.151	0.243	0.216

The NPEB was associated with the worst scores, thereby reflecting the nurses’ environmental behavior and activities in the workplace (52.23% rarely performed relevant activities, and 35.03% indicated that doing so was difficult) (Fig. 1 and Fig. 2). The NPEB values pertaining to environmental behavior were positively linked to age (*r* = 0.412; *p* < 0.001), NAS knowledge (*r* = 0.526; *p* < 0.001), PEB frequency (*r* = 0. 57; *p* < 0.001), PEB difficulty (*r* = 0.329; *p* < 0.001), and finally, difficulty performing adequate environmental behaviors (*r* = 0.499; *p* < 0.001). Additionally, the value of the NPEB with regard to the difficulty of performing adequate environmental behaviors was positively associated with age (*r* = 0.149; *p* = 0.008), NAS knowledge (*r* = 0.249; *p* < 0.001), PEB frequency (*r* = 0. 244; *p* < 0.001) and PEB difficulty (*r* = 0.442; *p* < 0.001).

Based on the relevance of certain sociodemographic variables, the nurses’ environmental awareness (NAS) and their behavior outside the workplace (PEB), linear multiple regression was performed to investigate nursing behavior in the workplace (NPEB). The initial model (square sum = 488.655; *p* < 0.0001) indicated that age, the impact of nursing awareness (NAS), and the frequency of sustainable behaviors outside the workplace (PEB) were not relevant to nursing behavior in the workplace (NPEB) in terms of the frequency of performing adequate behavior or the difficulties experienced (*p* > 0.05). Based on these results, the adjusted model was calculated (Table 3), indicating that NPEB depends on PEB frequency and NAS knowledge (*p* < 0.01).

**Table 3:** Linear multiple regression model with regard to nursing behavior in the workplace (NPEB) in terms of the frequency of performing adequate behaviors and the difficulties experienced

	Dependent variable (NPEB)	Square sum	Quadratic mean	F	p.
Adjusted model	Frequency	5326.225^a^	2663.113	107.500	< 0.001
	Difficulty	1028.690^b^	514.345	13.978	< 0.001
Intersection	Frequency	1045.803	1045.803	42.215	< 0.001
	Difficulty	6324.429	6324.429	171.872	< 0.001
PEB frequency	Frequency	1726.609	1726.609	69.697	< 0.001
	Difficulty	256.119	256.119	6.960	0.009
NAS knowledge	Frequency	1088.099	1088.099	43.923	< 0.001
	Difficulty	283.153	283.153	7.695	0.006
Error	Frequency	7704.428	24.773		
	Difficulty	11443.937	36.797		
Total	Frequency	191169.000			
	Difficulty	220031.000			

^a^ R Square = 0.411 (Adjusted R Square = 0.405)

^b^ R Square = 0.214 (Adjusted R Square = 0.206)

Nursing environmental behavior in the context of Green Teams: Barriers and areas for improvement.

The participants in the qualitative study (*N* = 10) included nine women and one man; their median age was 49 years; they exhibited an interval quartile range of 35–60; they had levels of working experience ranging between 20 and 30 years, and they worked only in the mornings (7/10). Furthermore, the group including nurses and nursing supervisors (5/10) exhibited higher levels of education (see Supplementary file 2). The themes identified via repetition and associations during the interviews and observations indicated links among nurses’ responsibilities on the Green Team since they conformed to the nature of such teams (i). This team and nursing leaders identified sustainable environmental behavior (ii) that could improve environmental awareness (iii), knowledge, aptitude, and skills. The nurses who are responsible for sustainable changes should be the leaders (iv), and the relevant barriers and limitations (v) and areas for improvement (vi) in diverse areas should be identified simultaneously.

(i) Green teams were linked to nursing responsibilities in the context of environmental sustainability.

In the interviews, the Green Teams, led by environmental leader nurses and comprising various staff members, were identified as crucial committees dedicated to enhancing environmental awareness and knowledge among hospital staff. Participants indicated that these teams facilitated regular meetings to discuss sustainable practices and played a pivotal role in testing behaviors and knowledge related to environmental sustainability. The Green Teams were highlighted as platforms for fostering collaboration and discussion surrounding sustainable practices. Participants noted that these teams facilitated the main purpose of the team and its members to improve the hospital staff’s knowledge and attitudes via meetings (RN 2,3 and NL 1,3). Subsequently, the NL also indicated a key role of the team in the testing of behaviors and knowledge. The behavior of registered nurses should be tested using questions according to the NLs. Also, the NLs are included in disponibility of of proper disposal methods for medical waste:

“So, where is the rubbish bin for medicines, that white one that you showed in the session that is used for the remains of medicines that we do not give to patients?” [(NL5)]

By such comments, it can be inferred that the Green Team not only disseminates information, manages the training and measures knowledge but also ensures that staff members understand and adhere to best practices in waste management. These tasks of the NLs and other RNs in the Green Team contribute to the overall efficiency and effectiveness of environmental sustainability efforts within the hospital.

(ii) Sustainable environmental behaviors were emerged by Green Teams.

The results of the analysis indicated some degree of resistance among the nurses working at the clinical management units with regard to their lack of competencies, especially those pertaining to knowledge, skills and attitudes. The comments from the interviews highlighted potential factors contributing to this resistance, including age-related differences, varying levels of awareness, and challenges in applying the principles of reduce, reuse, and recycle (the three Rs). For instance, one repetitive comment expressed a sentiment of uncertainty, stating “It is what is, but we don’t know it or what to do with it” (RN 3,4,5, and NL 2,3).

“We know what the light packing is, and they (maintenance people) installed it to reduce the lights and reduce the expense and cost, but we don’t know what to do with the rubbish bins” [(NL 4)]

This comment highlights a disconnect between awareness of specific sustainable initiatives and the practical knowledge to implement them effectively. All comments reflect the importance of addressing knowledge gaps and providing practical guidance to support nurses in adopting sustainable environmental behaviours. By acknowledging and addressing these challenges, healthcare facilities can enhance their environmental stewardship efforts and promote a culture of sustainability among staff members.

(iii) Environmental awareness were drawn from the nursing responsibilities that led to the creation of the Green Team.

The comments indicated that environmental awareness among nurses was influenced by training sessions and courses on environmental sustainability. After receiving training featuring lectures and courses on environmental sustainability, the leaders also reflected on the ways in which nurses put the recommendations made during the environmental sustainability courses into practice. Moreover, the leaders indicated that education should be beyond formal training sessions. The environmental leaders were interested in supplementing these courses with environmental education practices for the general population, as noted, for example, in reports of discharge from patient care or cycling on the ward. These activities indicated the ideal of including a holistic approach to sustainability within the healthcare setting.

Relevant statements included, “We have to separate residues according to the material… light plastic goes to… it is important for the unit and all of us” (NL 2,5). One key point that the referees and registered nurses highlighted pertained to the climate, particularly the lack of water (NL 1–5 and RN 1,2).

“The drought is getting worse; I don’t know how we are going to keep up… we hope it rains soon” [(RN1)]

Overall, the interviews shed light on the efforts to foster environmental awareness among nurses through formal training and practical integration into everyday practices. These observations emphasize the importance of ongoing education and action in addressing environmental concerns within healthcare settings.

(iv) Leadership, which was linked by comments to the Green Teams.

The interviews revealed that leadership, particularly within the context of Green Teams, is crucial in promoting environmental awareness and fostering a culture of sustainability among nursing staff. All the participants (*n* = 10) indicated that the presence of adequate knowledge, meetings and awareness among nursing staff were the most important factors. These factors were identified as key drivers in promoting sustainable practices within the healthcare environment. NLs indicated the importance of creating a supportive working environment where nurses feel comfortable asking questions and seeking clarification without fear of negative feedback. Relevant statements included, “It is key to receive feedback from the nurses and provide a good working environment so that they can ask questions and reflect without negative comments” (NL 1,2,4, and RN 1,2). This working environment allowed the registered nurses to ask for help regarding the three Rs:

“Could you remind me (referring to the environmental coordinator) how the sustainable guidelines were included in the discharge report for the continuity of care; I remember some things from the course you gave us, but I want to convey it completely to my patient” [(RN2)]

(v) Barriers and limitations, were drawn from nurses’ responsibilities.

Several nurses indicated that the difficulties they encountered with regard to performing environmental behaviors pertained to the lack of time, adequate bins, and space as well as the limited number of nurses per patient in the wards. Despite these challenges, participants noted a positive outcome in the form of increased awareness of sustainability issues among nurses, indicating a growing recognition of the importance of environmental stewardship within the healthcare setting. One factor that increased the barriers to environmental adequacy was the pandemic, which increased waste and rubbish. Despite these challenges, participants noted a positive outcome in the form of increased awareness of sustainability issues among nurses, indicating a growing recognition of the importance of environmental stewardship within the healthcare setting. Relevant statements included “There are not enough green rubbish bins for COVID waste” (EL 1,4,5 and RN1,2) and “How are we going to recycle if we don’t even have time to care for patients?” (RN 1,2 and NL 3).

All these comments indicated the barriers the nurses faced, but they also suggested possibilities for improvement. The pandemic, despite overloading nurses, also improved their awareness.

(v) Areas subject to improvement emerged from nursing responsibilities, limitations and leadership.

Nurses indicated that despite their general levels of environmental awareness and the courses they had received, participants performed better regarding their recycling behaviors at home than at the hospital. Participants acknowledged performing better in recycling practices within their personal spaces, suggesting a potential gap in translating theoretical knowledge into practical action within the healthcare environment. Relevant statements included “It’s just that I recycle almost everything in my house, especially glass…, but here, there is no time…” (RN 1,4,5).

Moreover, time constraints emerged as a significant barrier impeding nurses’ ability to engage fully in environmental sustainability efforts. Participants cited the demanding nature of their work, particularly in the context of patient care responsibilities, as limiting their capacity to prioritize sustainability initiatives. This highlights the need for strategies to streamline environmental practices and integrate them seamlessly into nurses’ daily routines without adding undue burden.

Some statements also highlighted nurses’ willingness to improve paperwork and records. Nurses recognized the importance of incorporating environmental considerations into patient discharge reports and other documentation processes but sought further guidance on how to effectively implement these practices. Relevant statements included “Can you tell me how the patient’s continuity care report upon discharge was included in the recommendations for environmental sustainability… I want to do the report well with what you gave us in the clinical session the other day…” [(NL4)]

These comments indicated the opportunities for improvement in fostering a culture of environmental sustainability within the hospital setting. By addressing the identified challenges and providing targeted support and guidance, especially the lack of time, nurses can contribute to environmental stewardship efforts more effectively.

## Discussion

The current research highlights the relevance of nurses as promoters of environmentally sustainable behaviors in their roles as members of Green Teams and important leaders. The findings suggest that nurses exhibit acceptable knowledge, attitudes, and behaviors with regard to environmental sustainability both inside and outside the workplace. These results are complemented by a qualitative analysis indicating that such behaviors originate from nursing responsibility, Green Teams, leadership identification of barriers and areas of improvement. Both analyses highlight the fact that environmental nursing behavior in the workplace depends on sustainable behaviors outside the workplace. The qualitative analysis also identifies diverse barriers to the task of promoting sustainable behavior within the workplace, such as the COVID-19 pandemic and the need for more time to be allocated to this process. One key point identified by both analyses is that nurses have acceptable levels of knowledge; however, their attitudes, although as yet imperfect, are improving.

Several studies of nurses’ awareness of environmental sustainability have revealed that nurses exhibit moderate levels of awareness and a considerable degree of concern regarding the health impacts of climate change [37, 42, 43], as reflected in the NEAT-es results.

Interestingly, the participants exhibited a tendency to perform environmentally sustainable behaviors more consistently in their personal lives than in professional settings. These results are consistent with previous research on registered nurse and nursing students [36, 41, 42]. According to Swedish research, nurses generally recognize environmental issues but may lack awareness of the environmental impact of health care [43]. Polivka Barbara J. et al. (2012) highlighted the gap between nurses’ knowledge of sustainability and workplace behaviors, thereby emphasizing the need for education and training programs to promote sustainable practices [44]. These issues were also observed in a study conducted in Taiwan, which revealed that while nursing students exhibit positive attitudes toward sustainability, their knowledge and behaviors are inadequate [45].

By conducting qualitative analysis, this research also identified multiple barriers to the adoption of sustainable practices among nurses, including time constraints, disruptions caused by the COVID-19 pandemic, a lack of bins, and a lack of health care personnel. These findings are in line with those reported in other research, but certain barriers (in terms of resources, time, and support) to the implementation of sustainable practices in the workplace remain [29]. This study suggests that interventions should be designed to address these barriers and promote sustainable behavior among nurses, a suggestion which is consistent with the current research. These findings highlight the importance of comprehending nurses’ perspectives on environmental sustainability in health care contexts as well as the necessity for targeted interventions and support mechanisms [46]. The tasks assigned to nursing leaders and the Green Team involved addressing these barriers and promoting sustainable practices among nurses in the context of their professional roles. Environmental nursing leaders seem to be crucial with regard to establishing a more environmentally conscious health care environment, which is in line with recommendations to create a greener health care system [21, 31]. Despite the results of the interviews, some global qualitative studies of nurses’ views on environmental issues have exhibited variations across countries [47, 48]. In Sweden, nurses already exhibit pro-sustainability attitudes before the introduction of the 2030 SDGs [16]. However, the integration of environmental sustainability education into nursing programs can prepare future nurses more effectively to address the challenges associated with climate change and promote sustainable health outcomes [49].

### Limitations

Although this investigation provides valuable insights, it is important to acknowledge its limitations. First, the study was conducted during the COVID-19 pandemic in Spain, which may have influenced the results due to the unique circumstances and stressors faced by health care workers during this period. Additionally, the assessment of nurses’ environmental awareness was performed on a larger scale, i.e., across multiple regions, and therefore may not accurately reflect individual attitudes and behaviors since the qualitative investigations focused on a specific region. However, this approach was adopted to minimize the risk of the ecological fallacy. Future studies could explore individual perspectives and experiences by reference to more diverse and representative samples.

Despite these limitations, this research is highly relevant because it sheds light on the role of nurses in the task of promoting environmental sustainability in health care settings. The research also emphasized the role of nursing leadership in the tasks of promoting environmental sustainability and providing nurses with the necessary resources and support to implement sustainable practices.

## Conclusion

In conclusion, while nurses generally exhibit acceptable levels of knowledge, attitudes, and behaviors regarding environmental sustainability, a notable gap persists in terms of the frequency of sustainable actions within the professional settings in which they operate. This finding highlights the importance of closely aligning nurses’ personal and professional sustainability practices.

The qualitative analysis conducted as part of this study identified several barriers to the adoption of sustainable practices among nurses, including time constraints, disruptions resulting from the COVID-19 pandemic, issues with waste disposal, and challenges related to health care personnel. Despite the fact that these findings are in line with those reported in previous research, persistent barriers such as limited resources, time, and support hinder the implementation of sustainable practices in the workplace. Therefore, interventions aimed at addressing these barriers and promoting sustainable behavior among nurses are essential, as highlighted by both current research and the corresponding qualitative insights. Therefore, nursing leaders and Green Teams are pivotal with regard to overcoming these barriers and fostering sustainable practices within health care environments. Environmental nursing leaders in particular are instrumental to the cultivation of a more environmentally conscious health care system, thereby aligning with recommendations for greener health care practices.

### Electronic supplementary material

Below is the link to the electronic supplementary material.

Supplementary Material 1

Supplementary Material 2

## Data Availability

The datasets used and/or analyzed as part of the current study are available from the corresponding author upon reasonable request.
