# Supplementary material for: The environmental awareness of nurses as environmentally sustainable health care leaders: a mixed method analysis
Source: BMC Nurs. 2024 Apr 3;23:229. doi: 10.1186/s12912-024-01895-z (PMC10988952; doi:10.1186/s12912-024-01895-z)
Supplement: Supplementary file 1 — Supplementary Material 1 [file 12912_2024_1895_MOESM1_ESM.docx]

**Supplementary file 1. The guide interview**

These questions were used as a guide for the interview in-person of the participants, being from general to specific and including mini-question for the conduction of the interview.

1. What do you think about the environmental crisis? And as a nurse and our role in the current situation?
2. Do you know the measurements in the unit available for recycling, waste disposal, and other containers?
3. Are enough containers and other tools for doing our part?
4. Are there measures for environmental sustainability, such as energy or water savings in the Hospital? Which ones are carried out in this unit?
5. Does the environmental reference nurse inform you and train you, solving doubts, for example, in online educational sessions? (Only for other nurses, not the reference nurses)
6. What do you think lacks of the nurses for implementing the protocol? (Only to the reference nurse)
7. What were the main barriers you encountered in implementing the ward's sustainable protocol?
8. Can the measures be improved to segregate waste, especially those increasing due to COVID?
9. What measures could be implemented to improve the implementation of environmentally sustainable protocol?
