# Supplementary material for: The environmental awareness of nurses as environmentally sustainable health care leaders: a mixed method analysis
Source: BMC Nurs. 2024 Apr 3;23:229. doi: 10.1186/s12912-024-01895-z (PMC10988952; doi:10.1186/s12912-024-01895-z)
Supplement: Supplementary file 2 — Supplementary Material 2 [file 12912_2024_1895_MOESM2_ESM.docx]

**Supplementary file 2.** Sociodemographic characteristics participants of the qualitative analysis

|  | **Frequencies**  **N %** | |
| --- | --- | --- |
| **Gender** |  |  |
| Female | 9 | 90 |
| Male | 1 | 10 |
| Non-binary | 0 | 0 |
| **Working experience** |  |  |
| More than 30 years | 2 | 20 |
| Between 21 y 30 years | 7 | 70 |
| Between 11 y 20 years | 1 | 10 |
| **Position held** |  |  |
| Register Nurse | 5 | 50 |
| Nursing Supervisor | 5 | 50 |
| **Ward** |  |  |
| Neurosurgery | 2 | 20 |
| Internal Medicine | 2 | 20 |
| Cardiology | 1 | 10 |
| Covid Unit | 3 | 30 |
| Others | 2 | 20 |
| **Shift** |  |  |
| Only morning | 7 | 70 |
| Rotating shift (switch between other shifts) | 3 | 30 |
